# Supplementary material for: Comprehensive Annotation and Functional Exploration of MicroRNAs in Lettuce
Source: Front Plant Sci. 2021 Dec 24;12:781836. doi: 10.3389/fpls.2021.781836 (PMC8739914; doi:10.3389/fpls.2021.781836)
Supplement: Supplementary file 2 [file Data_Sheet_8.ZIP › GSM2230747.plot/Lsa-miRN1652a_Lsat_1_v5_gn_5_101660.1_47_TPlot.pdf]

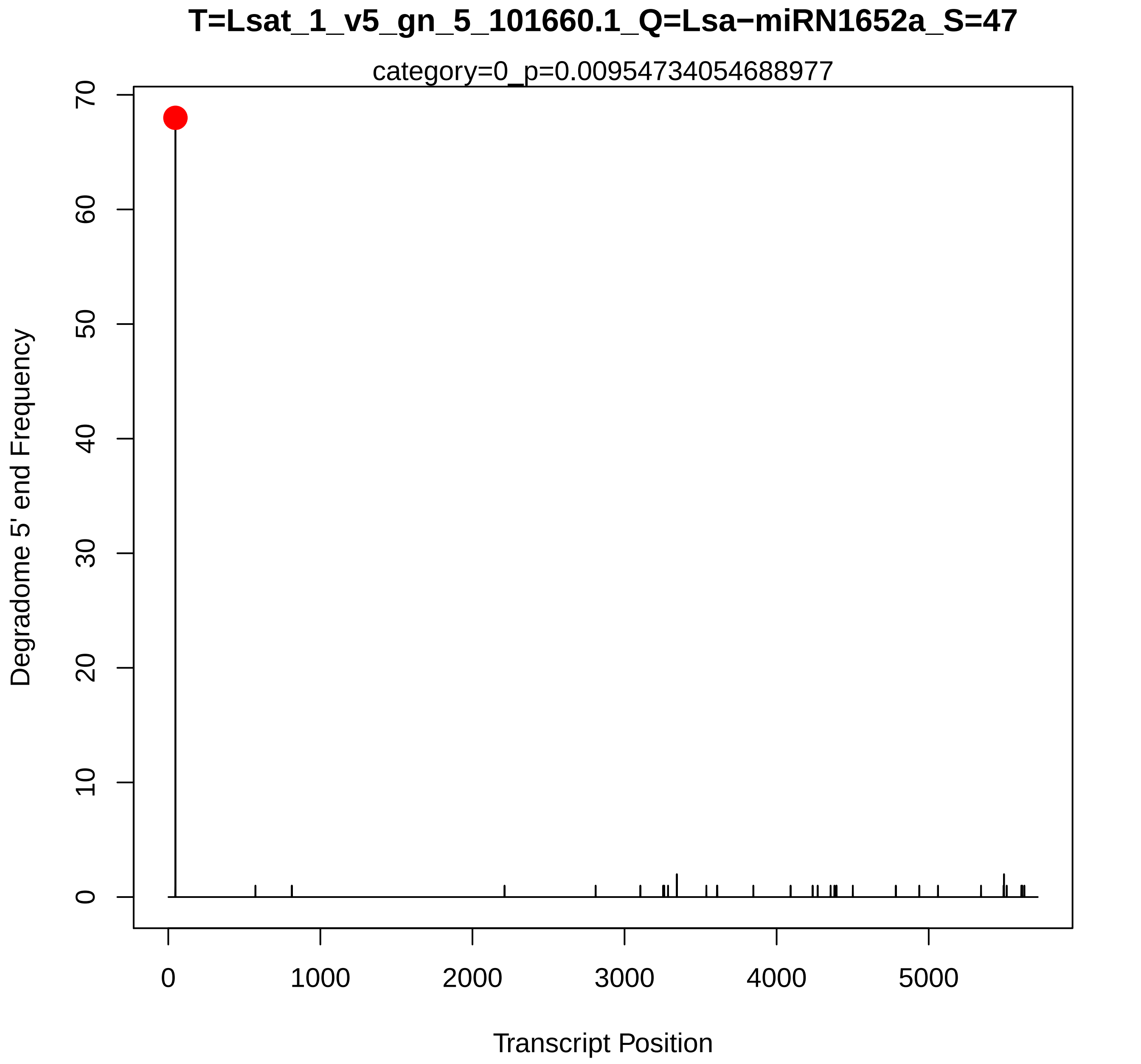

T=Lsat_1_v5_gn_5_101660.1_Q=Lsa−miRN1652a_S=47
category=0_p=0.00954734054688977
Degradome 5' end Frequency
0
10
20
30
40
50
60
70
0
1000
2000
3000
4000
5000
Transcript Position
